# Simulating Early Clinical Experiences With Surgical Procedures in the Anatomy Laboratory

**DOI:** 10.7759/cureus.13966

**Published:** 2021-03-18

**Authors:** Tayler Gant, Harrah Chiang, Benjamin D Harman, David S Axford, Paul Brisson, Michael Brisson, David Stephen

**Affiliations:** 1 Medicine, Edward Via College of Osteopathic Medicine, Auburn Campus, Auburn, USA; 2 General Surgery, Edward Via College of Osteopathic Medicine, Auburn Campus, Auburn, USA; 3 Epidemiology and Biostatistics, Edward Via College of Osteopathic Medicine, Auburn Campus, Auburn, USA; 4 Pathology, Edward Via College of Osteopathic Medicine, Auburn Campus, Auburn, USA

**Keywords:** surgical skills day, procedure training, skills and simulation training

## Abstract

Background

There is evidence to suggest that early exposure to clinical experiences could bolster a medical student’s education and prepare them to tackle the problem-based learning encountered during clinical rotations. We hypothesized that incorporating common surgical procedures into the gross anatomy laboratory during preclinical years would enhance the anatomical learning experience for students. The incorporation of these procedures would not be disruptive to the normal conduct of the anatomy laboratory, nor result in exorbitant costs.

Objectives

The goal of a more integrated curriculum is to better enable medical students by providing them a unique learning experience, so that they may more readily recall the knowledge needed to deal with the complex problems of clinical work. Recognizing the importance of this concept, we have incorporated multiple common clinical procedures into our anatomy curriculum as a pilot program.

Methods

Seven common surgical procedures, including intraosseous needle insertion, venous cut-down, chest tube insertion, surgical cricothyroidotomy, core needle liver biopsy, appendectomy, and hysterectomy, were taught as a part of this study. Video instructions on each of the surgical procedures were provided before each corresponding laboratory. Surveys were distributed to study participants to measure their satisfaction with the procedures and whether or not it was disruptive to the allotted laboratory time.

Results

Both students and faculty who were sampled in the study reported that they were satisfied with the procedures (96.5% and 100%, respectively), that the procedures did not interfere with laboratory times (96% and 100%), and that the procedures facilitated clinical learning (98% and 100%).

Conclusion

This study demonstrated that providing a novel surgical teaching program to medical students was beneficial to their education and non-disruptive to the conventional anatomy curriculum. This exercise further facilitates osteopathic education by demonstrating how structure and function organize surgical practice. The integration of Edward Via College of Osteopathic Medicine, Auburn Campus's (VCOM-AC’s) surgical procedures into other medical school anatomy courses can yield more prepared and confident students as they venture into their clinical rotations.

## Introduction

The need for incorporating surgical faculty into the traditional curriculum in the first two years of medical training was first identified in the 2004 Blue Ribbon Report to the American Surgical Association [1-3]. Following this identification came to a discussion highlighting the need for novel teaching techniques and learning opportunities [4,5]. The goal of a more integrated curriculum is to better enable medical students by providing them a unique learning experience, so that they may more readily recall the knowledge needed to deal with the complex problems of clinical work. Recognizing the importance of this concept, we have incorporated multiple common clinical procedures into our anatomy curriculum as a pilot program.

Various institutions reported similar strategies for only a single procedure involving hands-on experience with a cadaver, yet offered only as an elective with limited opportunity, and not incorporated into the medical education curriculum [5,6]. Edward Via College of Osteopathic Medicine, Auburn Campus (VCOM-AC) is the only medical institution integrating surgical procedures into the anatomy curriculum. Our initial aim was to determine if there was educational value in the incorporation of various procedures in the anatomy laboratory and if so, to provide a detailed blueprint for the expansion of our program and the potential initiation of similar programs at other medical schools. We propose that an anatomy curriculum including common surgical procedures amplifies medical students’ skill sets and knowledge of anatomical structures, thus improving preparation for clinical rotations.

## Materials and methods

Seven surgical procedures were chosen, for commonality and practicality, by a board-certified General Surgeon. They were to be conducted in a surgical setting which included access to materials and is to be completed in the cadaver laboratory within the first 15 minutes of the 90-minute allotted laboratory time. These surgical procedures included surgical cricothyroidotomy, intraosseous (IO) needle insertion, tube thoracostomy insertion, liver biopsy, hysterectomy, venous cut down of the great saphenous vein, and appendectomy.

This study was conducted at VCOM-AC where the four-year medical program consists of four blocks of the required cadaveric laboratory. Following IRB (2016-046) approval, the surgical procedures were incorporated into the anatomy laboratories involving relevant structures to the surgical procedure and the current learning objectives in the anatomy curriculum. The required equipment was funded through the VCOM-AC Department of Anatomy. Surgical procedures were scheduled so that they coincided with laboratories with relevant dissection objectives. For example, the appendectomy procedure was scheduled during the dissection of the small and large intestines and mesenteric vessels.

Instructional videos were created by a board-certified general surgeon faculty member and students were instructed to view the videos prior to the associated laboratory. The videos included indications, contraindications, complications, an overview of the relevant anatomy, and an expert demonstration of the surgical procedure. Medical students who were former or current paramedics provided an additional expert resource for certain procedures, such as the IO needle insertion. Procedural training of the anatomy biomedical faculty also proved to be an essential resource in the efficient progression of the procedural portion of the laboratory The general surgeon faculty member provided further instruction on the procedures in the anatomy laboratory, with the additional supervision of six trained non-clinical anatomy staff to assist the students throughout the procedures. The procedural faculty was supplemented at times with an ER physician for IO needle insertion, and an obstetrician (OB/GYN) physician for the hysterectomy procedures.

Every student in each anatomy dissection group, ranging from four to six students, participated in each procedure. The same group of students was followed through their one year of anatomy dissection, which encompassed three out of four blocks during the first year and the first block of the second year. Due to the limited availability of organs, procedures had to be modified so that every student could participate in the procedural learning. For example, since there is only one cricothyroid membrane, the students were instructed to perform additional tracheostomies between inferior cartilaginous tracheal rings. Similarly, with the absence of appendixes, the cadavers were set up so that the students could perform the steps of an appendectomy on an epiploic appendage in the right lower quadrant.

**Figure 1:**
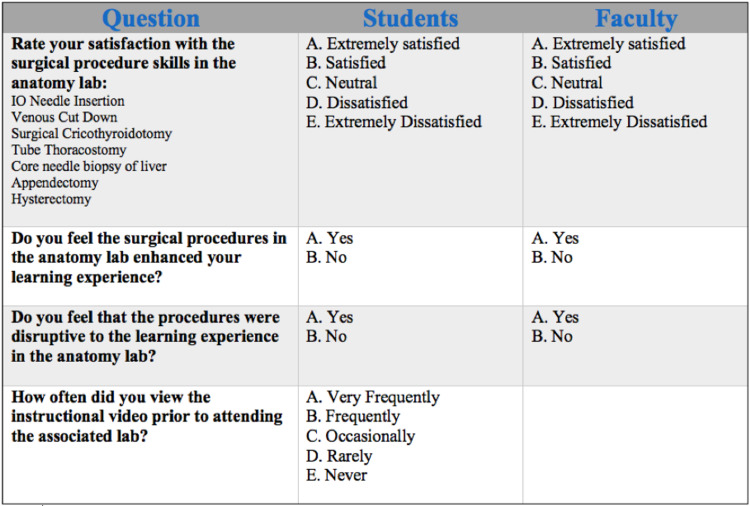
Survey Questions

Following the completion of the one-year project, both faculty members and students were asked to complete an anonymous online combined Likert and modified Likert scale survey regarding their experiences with these procedures. We designed the survey to determine the effect these procedures had on anatomical learning and to gage the degree of disruption to the scheduled curriculum of the anatomy laboratory (Figure 1).

The fourth question was only asked of the student sample. During our last mandatory anatomy lecture, the students were surveyed using an in-class electronic method, iClickers, to maximize participation. The faculty completed the first three questions via an online survey.

Each procedure did require the purchase of some equipment. Most of the purchased equipment was both expired and reusable. Some equipment did require replacement, such as tracheostomy tubes with balloon rupture, bent angiocatheters, and broken intraosseous needles. The equipment utilized for the study and the relevant costs is included in Table 1.

**Table 1:** Equipment Cost Per Procedure

Procedure	Equipment	Number	Cost/Item	Total
IO needle insertion	Power needle drivers	5	$425.00	$2,125.00
	Drill needles (expired)	10 sets	$40.00	$400.00
	IO hand-held needles	6		$75.00
Venous cut-down	2-0 silk suture spools, 25 yards	15	$14.00	$210.00
	Angiocaths (expired)	50	$10.00	$150.00
Surgical cricothyroidotomy	Tracheostomy tubes (expired)	15	$11.00	$165.00
	10 ml syringes, Leur lock,	30	$0.50	$15.00
	Disposable Airbag, BVM	15	$11.99	$179.85
Tube thoracostomy	Chest tubes (expired)	30	$10.00	$300.00
	Curved Kelly clamps	30	$10.00	$300.00
Core needle biopsy of liver	20 core needle biopsy instruments	20	$15.00	$330.00
Appendectomy	Babcock clamps	20	$15.00	$300.00
	Curved hemostats	30	$5.00	$150.00
Hysterectomy	Instruments			$200.00

## Results

The sample included 157 students and 11 anatomy faculty members. The survey was not mandatory, and 142/157 students responded to the first question of the survey, 137/157 students responded to the second question, and 144/157 students responded to the third and fourth questions of the survey. Ten of 11 faculty members responded to the first question, 9/11 responded to the second, and 10/11 responded to the third question of the survey. All values presented in the results section are in respect to the number of students and faculty members who participated in the survey.

**Figure 2:**
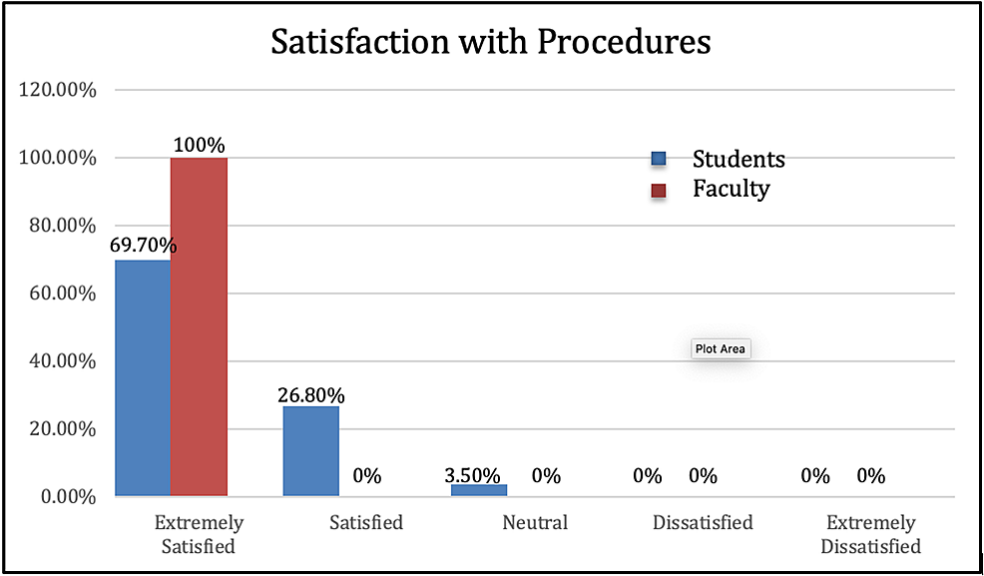
Student Satisfaction

For nominal data, the frequency with which each category was selected, as well as the percentage of responses accounted for by each category, is shown in Figure 2.

A total of 69.7% of students (n=99) indicated that they were extremely satisfied with the surgical skills training, and 26.8% (n=38) were satisfied. Further, 3.5% reported being neutral (n=5) and no students reported being dissatisfied nor extremely dissatisfied (Figure 2).

**Figure 3:**
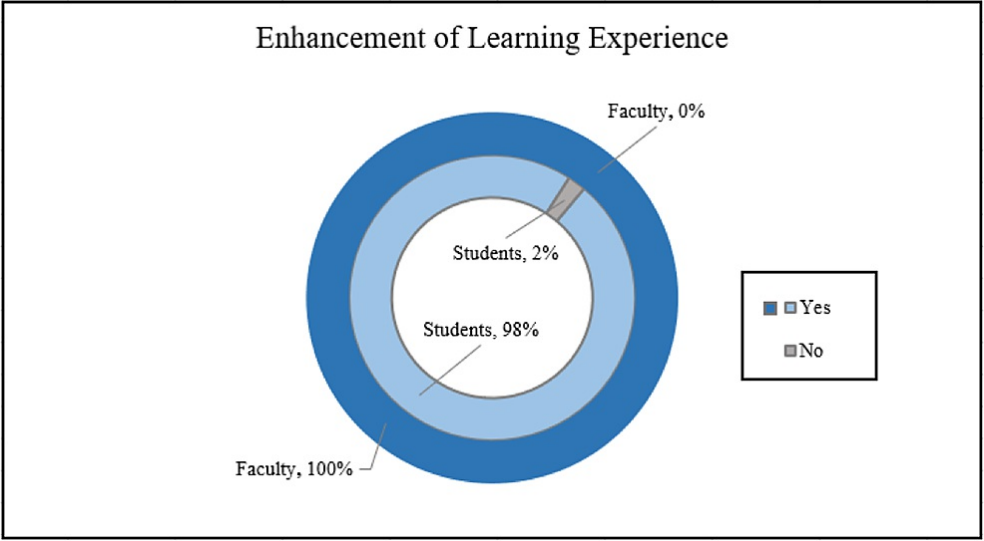
Enhancement of Anatomy Learning Experience

The students were asked if the surgical skills training enhanced their anatomy laboratory learning experience, to which 98.0% (n=134) responded yes and 2% (n=3) responded no (Figure 3).

**Figure 4:**
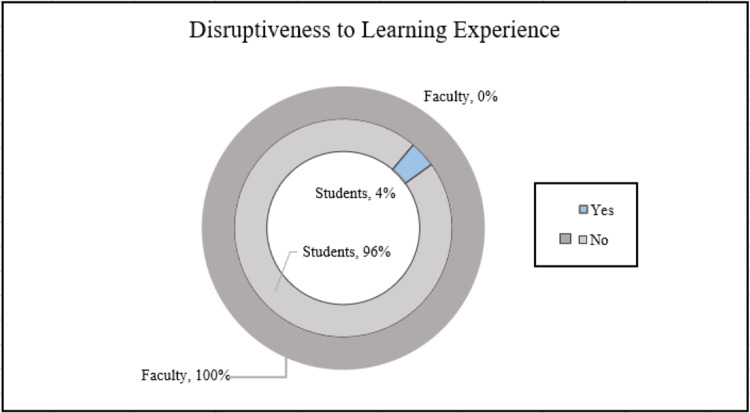
Disruptiveness to Anatomy Learning Experience

Students were also asked if the surgical skill training sessions were disruptive to the overall anatomy learning experience (Figure 3). 96% (n=138) of students indicated that the procedures were not disruptive while 4% (n=6) felt that it was disruptive (Figure 4). 

**Figure 5:**
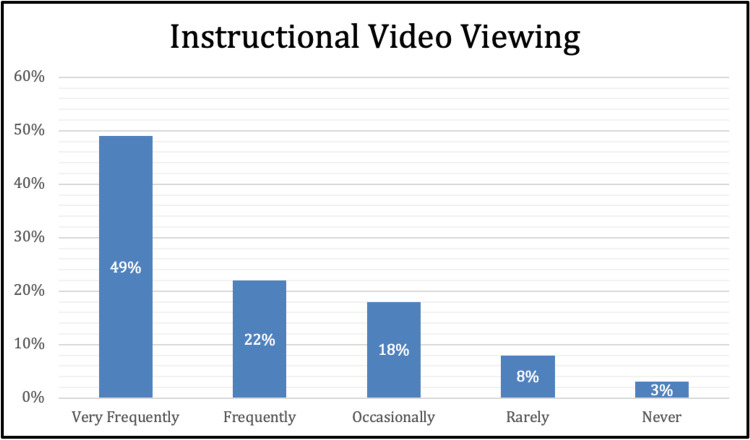
Instructional Video Viewing

 The final survey question inquired if the students watched the instructional video prior to the laboratory. Forty-nine percent of students (n=70) responded as very frequently, 22% (n=32) responded as frequently, 18% (n=26) occasionally, 8% (n=12) rarely, and 3% (n=4) never watched the videos (Figure 5).

A total of 100% (n=10) of faculty members reported extreme satisfaction with the surgical procedure skills (Figure 2). No faculty member reported being satisfied, neutral, dissatisfied, or extremely dissatisfied. When faculty members were asked if the surgical procedures enhanced the anatomy laboratory learning experience for the first-year and second-year medical students, 100% (n=9) reported that the procedures did enhance the learning experience (Figure 3).

When asked if the surgical procedural skills disrupted the anatomy learning experience, 100% (n=10) of faculty members reported that it was not disruptive (Figure 4).

## Discussion

There is an increasing need for new physicians to become more competent in basic surgical skills as they move on to their postgraduate training [7-9]. Authors have commented on the need for alternative teaching strategies to provide medical students with the opportunity to develop their own tactics to retain large amounts of information, develop critical thinking skills, and prepare them for complex clinical situations [10,11].

We hypothesized that incorporating surgical procedures into the anatomy laboratory during the preclinical years would enhance the student learning experience and would not be disruptive to the anatomical learning experience.

The survey demonstrated that students and faculty deemed surgical procedure instruction in the anatomy laboratory both beneficial and non-disruptive to the learning experiences. We expected a favorable response to be reflected in the survey from the medical students. However, we did not anticipate such a positive response from the faculty, since the surgical procedures could have infringed on their time to fulfill the learning objectives for the anatomy laboratories. The general assent from the anatomy faculty may have been related to several factors, including the enthusiasm of the students, the small amount of time required for each procedure, and faculty procedural training for this unique program that enhances their own skills and knowledge.

Some unanticipated benefits of the program included the discovery that the cadaver lungs can be visibly ventilated when a bag-valve-mask is attached to the tracheostomy tube. Each student viewed the expansion and contraction of the lungs as they ventilated their respective cadaver. Furthermore, since suture tying was a component of the appendectomy procedure, most students practiced and improved their knot-tying skills prior to and during the procedure. These additional gains foster an osteopathic education through the direct visualization of how anatomical structures can be manipulated during surgery to influence its function.

The strengths associated with the design of this study include having a large number of participants and high response rates to the surveys. Some of the limitations of this project are the minimal time allotted to learn each procedure, the lack of surgically viable organs, the non-mandatory survey participation for students, the non-mandatory viewing of the instructional videos, the brief survey questionnaire, the differences between cadaver tissue and living human tissue, and that the results were collected from a single institution. The optional nature of the survey is likely why there was a variable response count for each question. 

Our project was also intended to develop a guide for the expansion of our own program and a blueprint for other medical schools that may wish to implement such a program into their curriculum. The instructional videos and anatomy laboratory dissection objectives could be compiled into a manual and used to develop a foundation for other medical institutions to possibly enhance their own curriculum.

## Conclusions

Researchers have proposed the need for integrated clinical learning exercises to enhance medical students’ capacity for clinically relevant skills during their preclinical and clinical training. We introduced a pilot program that is unique to anatomical education where medical students performed seven surgical procedures in the anatomy laboratory, each relevant to the current systems-based area of study and clinical medicine. Resources included a general surgeon, an OB/GYN, an ER physician, surgical equipment, instructional videos, and brief training of the biomedical faculty. The procedures were constricted to the first fifteen minutes of laboratory time, including demonstration and participation from each student. A vast majority of students and faculty members reported that the program enhanced the learning experience without disruption of normal anatomic learning. Our research has provided support and guidelines for the potential expansion to other medical schools interested in incorporating surgical procedures into their anatomy curriculum.
